# Supplementary material for: 880 nm NIR-Triggered Organic Small Molecular-Based Nanoparticles for Photothermal Therapy of Tumor
Source: Nanomaterials (Basel). 2021 Mar 18;11(3):773. doi: 10.3390/nano11030773 (PMC8003086; doi:10.3390/nano11030773)
Supplement: Supplementary file 1 [file nanomaterials-11-00773-s001.pdf]

nanomaterials

Supplementary Materials

880 nm NIR-Triggered Organic Small Molecular-Based Nanoparticles for Photothermal Therapy of Tumor

Yunying Zhao [1,†], Zheng He [1,†], Qiang Zhang [1], Jing Wang [1], Wenying Jia [1], Long Jin [1], Linlin Zhao [1,2,*] and Yan Lu [1]

[1] School of Materials Science & Engineering, Tianjin Key Laboratory for Photoelectric Materials and Devices, Key Laboratory of Display Materials & Photoelectric Devices, Ministry of Education, Tianjin University of Technology, Tianjin 300384, China; 17853483674@163.com (Y.Z.); 13752723282@163.com (Z.H.); zhangqiang@email.tjut.edu.cn (Q.Z.); wangjing@iccas.ac.cn (J.W.); jia15249238411@163.com (W.J.); KimYong0205@163.com (L.J.); luyan@tjut.edu.cn (Y.L.)

[2] State Key Laboratory of Molecular Engineering of Polymers, Fudan University, Shanghai 200433, China

* Correspondence: linlinzhao@email.tjut.edu.cn

† These authors contributed equally to this work.

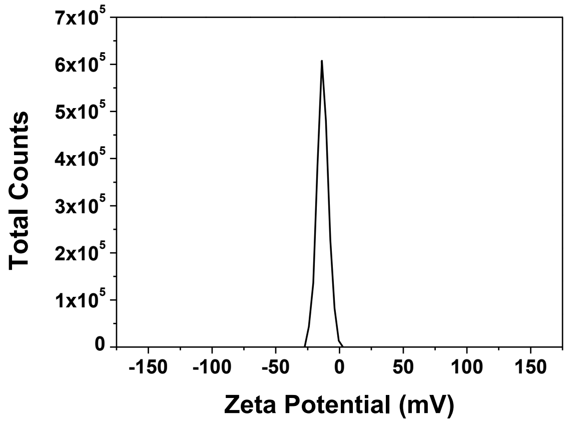

Figure S1. Zeta potential of TNPs in water.

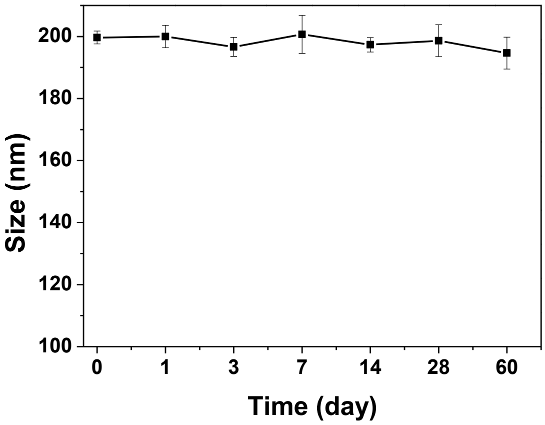

Figure S2. Changes of hydrodynamic diameters of TNPs in DMEM with time, [TNPs] = 180 μg/mL.

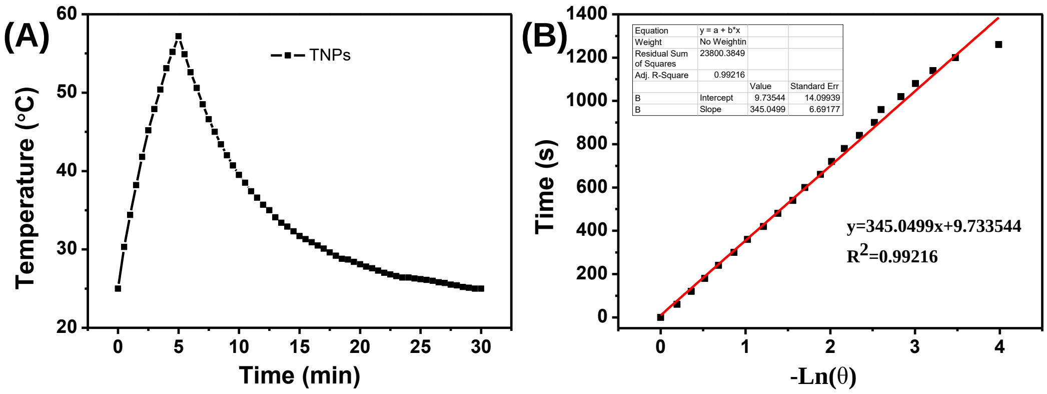

Figure S3. (**A**) Temperature elevation of TNPs (180 μg/mL) under 880 nm irradiation at 0.7 W/cm² for 5 min, followed by subsequent cooling to room temperature and (**B**) Linear time data versus-Ln (θ) obtained from the cooling period of NIR laser off.
